# The adverse and beneficial effects of polyphenols in green and black teas *in vitro* and *in vivo*

**DOI:** 10.7150/ijms.85521

**Published:** 2023-08-15

**Authors:** Mei-Hui Wu, Jer-Yuh Liu, Fang Ling Tsai, Jyuan-Jen Syu, Ciao-Sin Yun, Liang-Ying Chen, Je-Chiuan Ye

**Affiliations:** 1 Department of Nursing, Tzu-Chi University of Science and Technology, Hualien, Taiwan; 2 Graduate Institute of Biomedical Sciences, China Medical University, Taichung, Taiwan; 3 Center for Molecular Medicine, China Medical University Hospital, Taichung, Taiwan; 4 Department of Bachelor's Degree Program for Indigenous Peoples in Senior Health and Care Management, National Taitung University, Taitung, Taiwan; 5 Master Program in Biomedical Science, National Taitung University, Taitung, Taiwan

**Keywords:** black tea, green tea, RSC96 cells, H9c2 cells, toxicity, protective effects, colorectal colitis.

## Abstract

Although numerous studies highlight the health benefits of tea, excessive consumption has been linked to toxic conditions. Thus, understanding the optimal consumption of tea is essential to minimize toxicity while maximizing its benefits. In this study, we investigated the effects of eight green tea samples (G1-G8) and eight black tea samples (R1-R8) from *Camellia sinensis*, the most popular teas in Asian culture, on RSC96 Schwann neural cells and embryonic cardiomyocyte H9c2 cells. The results showed that the IC_50_ (mg/ml, weight/volume) of both tea types were inversely proportional to their polyphenol content, suggesting a relationship between toxicity and polyphenol levels in both green and black tea. Interestingly, green teas generally have higher polyphenol content than black teas. We also assessed the protective effects of tea *in vitro* by pretreating cells with the teas at indicated doses of polyphenol and subsequently exposing them to H_2_O_2_. Both tea types significantly reduced the decline in cell viability for both cell lines, and there was no significant difference in protective polyphenol concentrations for green (G3 & G7) and black (R3 & R8) teas at effective concentrations (EC20 and EC40). To evaluate the preventative effects of tea *in vivo*, we examined the impact of two green (G3 & G7) and two black (R3 & R8) teas with varying polyphenol content on dextran sulfate sodium (DSS)-induced inflammatory colitis in mice. Tea-treated groups exhibited significantly lower inflammatory scores (DAI) than the control group. DSS treatment in the control group led to shortened colorectal lengths in mice, while tea co-treatment partially prevented this loss. Histological analysis revealed that G7 and R3 (with a moderate polyphenol content) treatment improved colorectal crypt structure, decreased the severity of inflammatory ulcerative colitis, and significantly reduced histological scores compared to the control group. However, G3 and R8 (with high and low doses of polyphenol content, respectively) did not show these effects, suggesting that a moderate polyphenol level in both tea types is optimal for preventative benefits.

## Introduction

Tea has long been an essential aspect of Asian culture, particularly in Japan and China, and is known for its unique physiological benefits 1. Adult and Elderly especially like to drink tea. The most widely consumed teas are green and black tea, both derived from the *Camellia sinensis* tea tree. The primary difference between these two types of tea is that black tea is fermented before drying and roasting, while green tea remains unfermented. Both teas contain natural polyphenols, such as caffeine and catechins (catechin (C), epicatechin (EC), epicatechin gallate (ECG), epigallocatechin (EGC), and epigallocatechin gallate (EGCG)) 2. Green tea can have up to 5% of EGC and EGCG content, whereas black tea has a lower total polyphenol content. Recent epidemiological studies have demonstrated an inverse association between risks, such as cancer, cardiovascular disease, and psychological dysfunction, and the consumption of polyphenol-rich foods and beverages 3-8, and have been mentioned on their antioxidant and anti-inflammatory properties 9.

However, according to reviews, consuming beyond the recommended safe level of polyphenols in teas can lead to hepatotoxic and gastrointestinal disturbances, such as vomiting and diarrhea 10-15. EGCG exhibits some neurotoxicity 16 and affects human and rat neural progenitor cells (NPCs) development *in vitro* 17. The underlying reasons remain unclear, but it is possible that polyphenol-rich beverages like green and black tea produce fairly high concentrations of H_2_O_2_ during preparation and storage at room temperature 18. As exogenous agents, polyphenols produce nM concentrations of H_2_O_2_ in cells and organs, activating signaling factors and increasing cellular Eustress 19. When polyphenols reach high concentrations in the bloodstream, around μM concentrations of H_2_O_2_, they induce cytotoxicity and stress due to their exaggerated pro-oxidative effect. High levels of catechin, a major polyphenol in green tea, have been found to enhance carcinogen-induced carcinogenesis 20, 21 and promote DNA cleavage in the presence of Cu^2+^ *in vitro* 22. Another study reported that EGCG was protective to DNA at low concentrations but exacerbated DNA oxidative damage at higher concentrations, displaying pro-oxidative effects on DNA 23. Taken together, we theorized that there is margin of effective polyphenol concentration that serves the purpose of maintaining redox homeostasis in the body ideal for conferring better health. Therefore, it is urgent to find the amount of tea that is both safe and conveys adequate protective effect.

Considering that the tea leaf production process, specifically the fermentation level, affects polyphenol content, it is hypothesized that consuming green or black tea at a suitable polyphenol level can generate beneficial H_2_O_2_ concentrations and protect the body from environmental toxins. In this research project, the toxic and protective effects of green and black tea were investigated *in vitro* using RSC96 Schwann neural cells and embryonic cardiomyocyte H9c2 cells to determine their effects on cell viability. Furthermore, the preventive effects of green and black tea were evaluated *in vivo* using a mouse model, examining their impact on dextran sulfate sodium (DSS)-induced inflammatory colitis.

## Materials and Methods

### Tea Extract Preparation

Sixteen commercial tea samples, consisting of eight green tea samples (G1-G8) and eight black tea samples (R1-R8) from central Taiwan, were collected, all from the species *Camellia sinensis*. Each 10 g tea sample was immersed in 100 ml boiling distilled water for 10 min. The leaves were separated using filtration, and the total extracts were adjusted to 100 ml. These final extracts were sterilized at 121 °C for 20 min and stored at 4 °C in a closed container, designated as 100 mg/mL (tea/water) for subsequent experiments.

### Polyphenol Content Assessment

The method employed by Singleton et al. 24 was utilized to measure polyphenol content. An extract (1 ml) was mixed with distilled H_2_O (10 ml) and Folin-Ciocalteu reagent (0.5 ml), shaken, and left at room temperature for 15 min. Around 3 ml of 20% Na2CO3 was added, and the solution was heated at 100°C for 1 min in a water bath. Using a spectrophotometer at 725 nm, the polyphenol content was calibrated to a gallic acid standard, and the percentage of polyphenol in crude leave extracts of both types of tea is shown in Table 1.

### Cell Culture

RSC96 cells were obtained from the Bioresources Collection and Research Center, and H9c2 myocardial cells were acquired from the American Type Culture Collection (ATCC; Rockville, MD). Cells were cultured in Dulbecco's modified Eagle's medium, supplemented with 10% v/v fetal bovine serum (Gibco BRL, Gaithersburg, MD, USA) and 100 μg/mL penicillin/streptomycin (Sigma-Aldrich Chemie, Munich, Germany) at 37◦C in a humidified atmosphere containing 5% CO2. The cells were seeded in 24-well culture plates at an initial density of 2 × 10^5^ cells/mL and grown to around 80% confluence. Oxidative stress was induced using fresh H_2_O_2_. The cells were pretreated with tea extracts at the indicated concentrations for 24 h, and the medium containing H_2_O_2_ (final concentration at 5.6%) was added and incubated for the indicated time periods. Morphological analysis was performed by observing cell size and number changes under an inverted microscope (Olympus Corp., Japan) at 100× magnification.

### MTT Assay

An MTT assay was used to determine cell viability. RSC96 or H9c2 cells were exposed to H_2_O_2_, with or without test sample pretreatments (teas). To establish hydrogen peroxide cytotoxicity, RSC96 or H9c2 cells were treated with five H_2_O_2_ concentrations, as indicated in a pilot study. This H_2_O_2_ concentration resulted in more than 30% cell death after 6 h compared to untreated control cells and was deemed suitable for subsequent experiments. RSC96 or H9c2 cells were starved for 6 h and pretreated with various indicated concentrations of teas for 24 h and treated with H_2_O_2_ for 24 h. After treatment, the medium was removed, and RSC96 or H9c2 cells were incubated with MTT 0.5 μg/mL at 37 °C for 4 h. Viable cell numbers were directly proportional to formazan production, dissolved in isopropanol, and determined by measuring absorbance at 570 nm using a microplate reader.

### Animals

All animal protocols adhered to animal care guidelines reviewed and approved by the Institutional Animal Care and Use Committee (IACUC) at China Medical University. Male BALB/c mice (18-20 g upon arrival, approximately 5 weeks of age) were procured from the National Laboratory Animal Center (Taipei, Taiwan) and acclimatized for 5-6 days before experimentation. Mice were individually housed in a room with a 12-h dark:light cycle and provided with mouse chow and drinking water ad libitum. Sample size was based on IACUC of China Medical University requirements.

Twenty-four male BALB/c mice (5 weeks old) were maintained under constant conditions (room temperature 23 ± 1°C, 12 h light/dark cycle) with sterilized reverse osmosis water and diet available ad libitum. The mice were divided randomly into six groups: the vehicle group, control DSS group, G3 (G3 + DSS) group, G7 (G7 + DSS) group, R3 (R3 + DSS) group, and R8 (R8 + DSS) group. Starting on day 0, 200 μl of G3, G7, R3, or R8 tea solutions (30, 30, 60, or 30 μl respectively of tea extract + water) (with 3.9, 2.1, 2.1, or 0.5 mg/kg of polyphenol levels, respectively) were administered daily via oral injection until the experiment's end (Fig. 3A) 25**.** On Day 5, the mice began receiving 3% DSS in drinking water for 7 days, as described in the previous study 26. Body weight, hematochezia, and stool characteristics were monitored according to the disease activity index [DAI = (Weight loss score + Stool characteristics score + Hematochezia score)/3] as described 27. On day 12, all mice were euthanized by CO_2_ asphyxiation. The colons were removed, and length was measured. The distal colons were fixed in a 10% formalin solution, embedded in paraffin, and stained with H&E according to standard protocols. Histological scoring was performed as described 28. Severe inflammation was diagnosed as ulcerative colitis (UC). A combined score of inflammatory cell infiltration (score, 0 - 3) and tissue damage including the large-scale degeneration and death of epithelial cells in the mucosal layer, fibrous tissue proliferation, goblet cell depletion, and crypt loss (score, 0 - 3) were added to give a histological score of 0 (no changes) to 6 (extensive cell infiltration and tissue damage).

### Hematoxylin-Eosin (HE) Staining and Histological Analysis

Paraffin-embedded colon tissues were sliced into sections (4 μm), which were deparaffinized and rehydrated through a xylene-ethanol-water gradient system. Hematoxylin and eosin (HE) staining was performed, followed by a dehydration process. Histopathological examination was conducted using a light microscope manufactured by Leica (LAS Software Version 4.9).

### Statistical Analysis

All parametric data are presented as mean ± SEM. For single time point comparisons, a two-tailed unpaired Student's t-test (two groups) or one-way analysis of variance (ANOVA) with Tukey post-hoc test (more than 3 groups) was conducted. For all statistical tests, a significant difference was considered at p < 0.05.

## Results

### The toxicity of green and black teas *in vitro*

**Figure 1:**
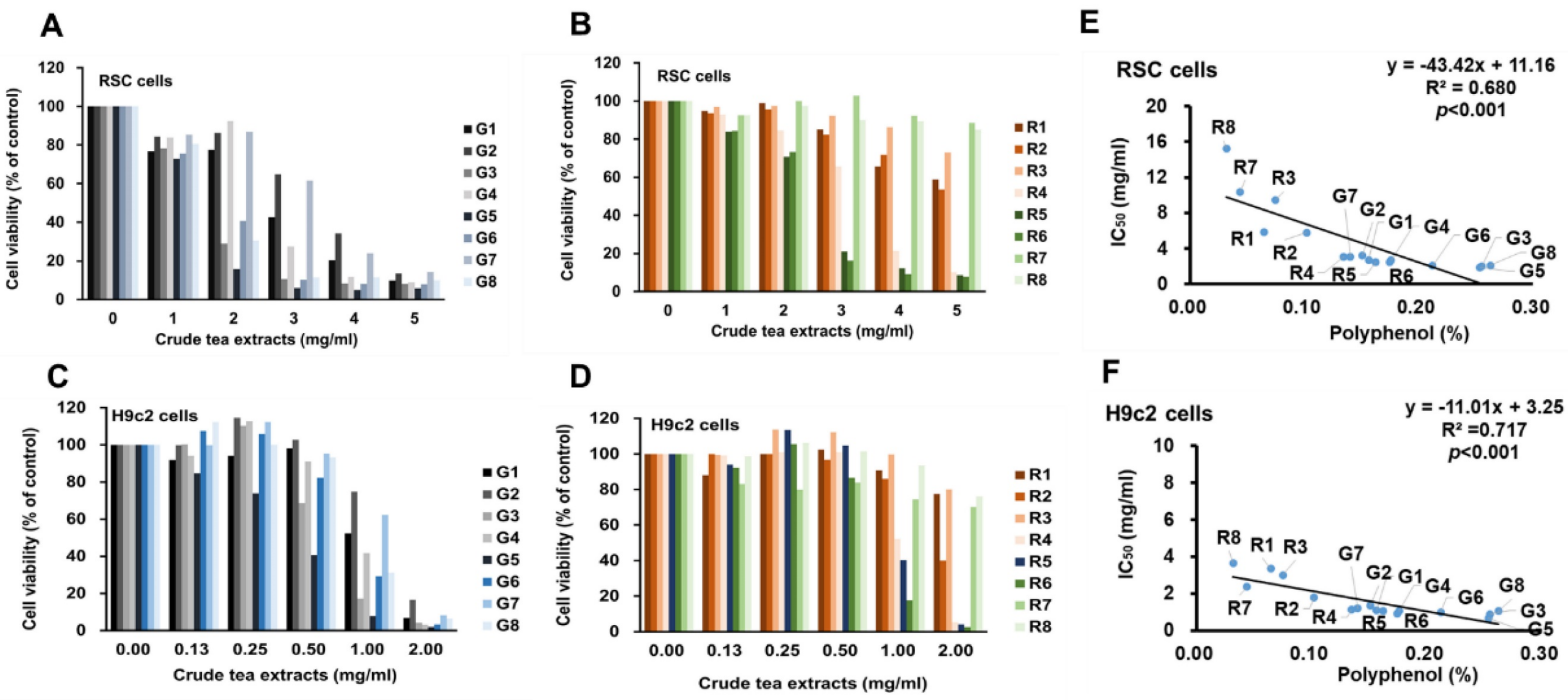
The toxicity of green and black teas *in vitro*. The RSC96 cells were treated with 1, and 2, 3, 4, and 5 mg/mL of green (A) and black (B) teas and H9c2 cells were treated with 0.125, 0.25, 0.5, 1, and 2 mg/mL of green (C) and black (D) extracts for 72hs. In the linear regression analysis, the levels of tea polyphenol contents in the each tea sample (5 g/100 ml) are significantly correlated with each IC_50_ concentration in both RSC96 (E) and H9c2 (F).

Toxicity test on RSC96 cell viability with 0.0, 1.0, 2.0, 3.0, 4.0, and 5.0 mg/ml of tea extracts for 72 hs showed that all green teas significantly decreased cell viability from 2 mg/ml in a dose-dependent manner (Fig. 1A), and two of the eight black teas resulted in the decrease of cell viability from the dose of 3.0 mg/ml in a dose-dependent manner (Fig. 1B). Toxicity test on H9c2 cell viability with 0.0, 0.125, 0.25, 0.5, 1.0, and 2.0 mg/ml of tea extracts for 72 hs showed significantly decreased cell viability from 0.5 mg/ml in a dose-dependent manner (Fig. 1C), and three of eight black teas significantly decreased cell viability from the dose of 1.0 mg/ml in a dose-dependent manner (Fig. 1D). In the linear regression analysis, the levels of tea polyphenol contents showed significant inverse correlation with each IC_50_ concentration (mg/ml, weight/volume) in both RSC96 and H9c2 cells (Fig. 1E and 1F). When IC_50_ was set to polyphenolic concentration (% of polyphenol, pIC_50_), the values of the green and black teas were at 0.0095 ± 0.0009 and 0.0096 ± 0.0023 for RSC96 cells respectively and 0.0040 ± 0.0007 and 0.0033 ± 0.0009 for H9c2 cells respectively (Table 1). Since reaction of cells to toxicity in the two types of tea were not significantly different (p < 0.05) in either cell types, this indicates the dependence of toxicity on polyphenol content.

### The protective effects of green and black teas* in vitro*

**Figure 2:**
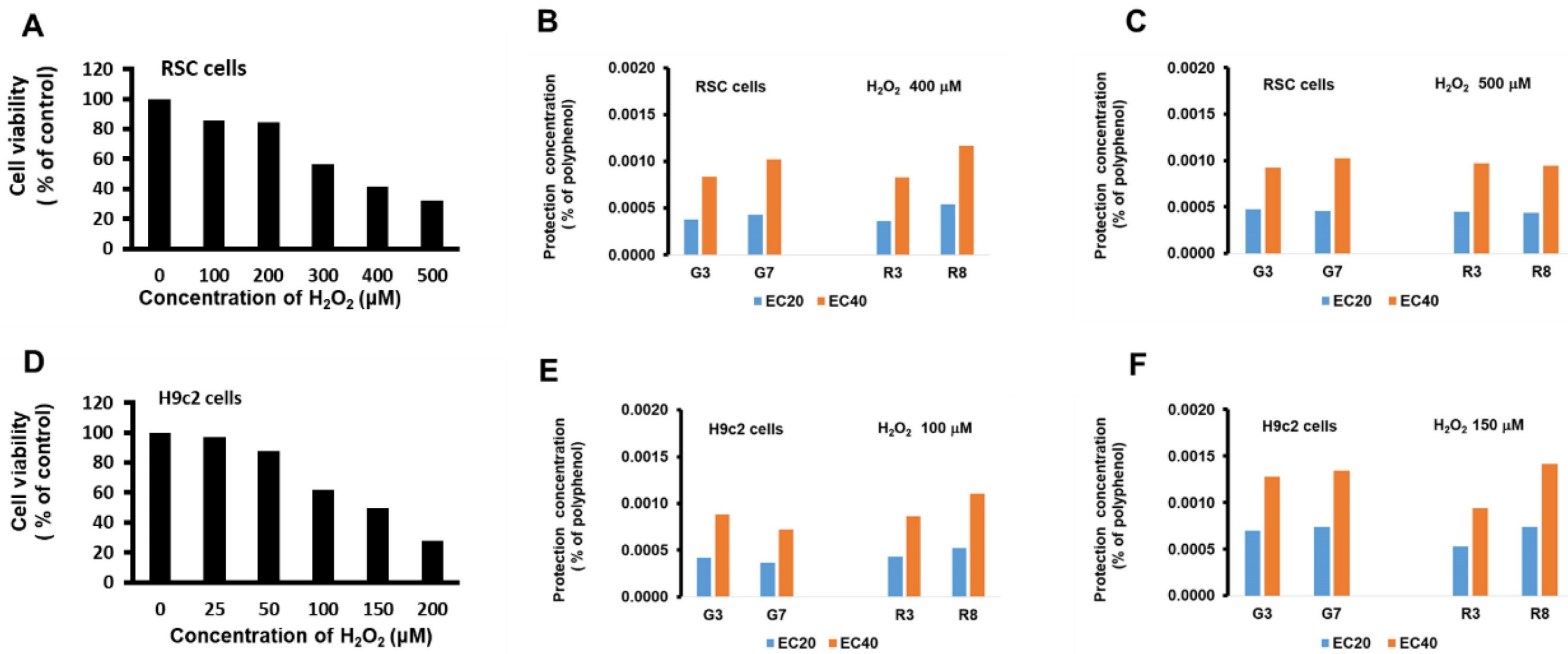
The protective effects of green and black teas *in vitro*. (A) RSC96 cells were treated with 0, 100, 200, 300, 400, and 500 μM H_2_O_2_ for 72 hs. Cells were pretreated with 0%, 0.0003%, 0.0006%, 0.0012%, and 0.0024 % polyphenol of teas for 24 hs and then challenged with 400 (B) or 500 (C) μM H_2_O_2_. The protection concentration of teas reversed the cell decreased viability in the administration of H_2_O_2_ by approximately 20% (EC 20) or 40% (EC 40). (D) H9c2 cells were treated with 0, 25, 50, 100, 150, and 200 μM H_2_O_2_. Cells were pretreated with 0%, 0.0003%, 0.0006%, 0.0012%, and 0.0024 % polyphenol of teas for 24 h and then challenged with 100 (E) or 150 (F) μM H_2_O_2_.

To examine the effects of the teas on H_2_O_2_-induced RSC96 cell death, we selected two of each type of teas with the highest and lowest polyphenol content (G3, G7, R3, or R8) for pretreatment for 24 hs before challenging with H_2_O_2_ for 72 hs. Administration of H_2_O_2_ decreased cell viability in a dose dependent manner, and 400 and 500 μM significantly diminished RSC96 cell viability by 41%, and 32% (Fig. 2A), respectively, and these concentrations were then subjected to EC20 (20% maximal effective concentration) and EC40 (40% maximal effective concentration) tests. The results showed that all tea types, when concentrations were adjusted to 0.0004 ± 0.0001% (Fig. 2B), were able to achieve EC20, and when concentrations were adjusted to 0.0010 ± 0.0001%, able to achieve EC40 (Fig. 2C).

On H9c2 cells, identical treatment also decreased cell viability in a dose dependent manner, and 100 and 150 μM significantly diminished H9c2 cell viability by 62%, and 50%, respectively. EC20 and EC40 tests show that all tea types, when concentrations were adjusted to 0.0006 ± 0.0002% (Fig. 2E), were able to achieve EC20, and when concentrations were adjusted to 0.0011 ± 0.0003%, were able to achieve EC40 (Fig. 2F).

A comparison of the green and black teas (G3, G7, R3 & R8) revealed no significant difference in protective polyphenol concentrations (EC20 and EC40) between these teas in both RSC96 and H9c2 cells. As EC20 and EC40 denote effective concentrations for cell protection, this suggests that the beneficial properties of these teas can be attributed to polyphenol concentrations ranging from 0.0004% to 0.0011%. In an animal study, for instance, 200 μl of G3, G7, R3, or R8 tea solutions (with polyphenol levels of 3.9, 2.1, 2.1, or 0.5 mg/kg, respectively) were administered via oral injection to mice. This study assumed that the effects observed would be similar to those in the previously mentioned study 25. Based on these findings, the investigation of the protective effects *in vivo* will proceed using the same dosage for subsequent experiments.

### The protective effects of green and black teas* in vivo*

**Figure 3:**
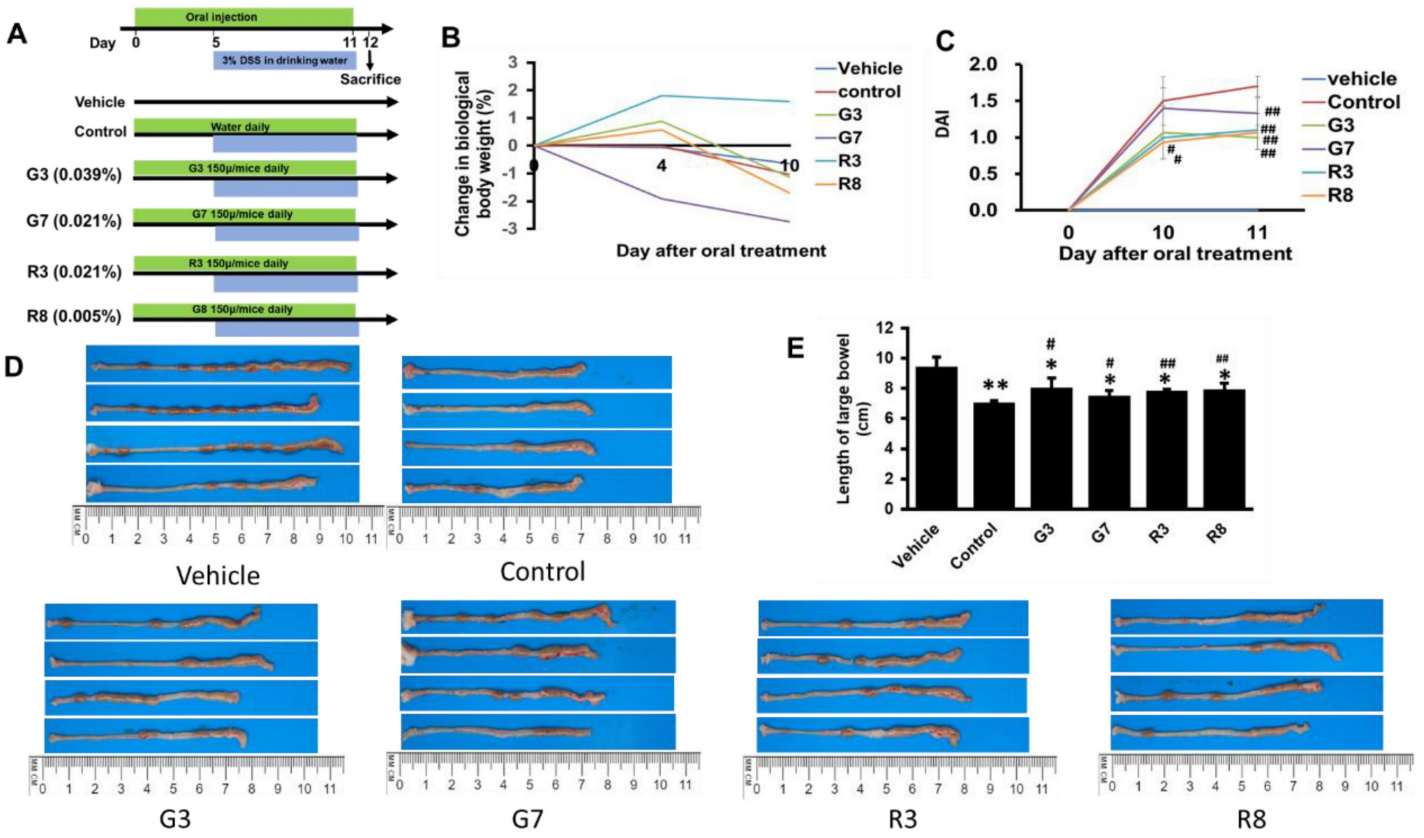
Teas reduced colitis incidence in DSS-treated mice. (A) Design of the experimental procedure. (B) Quantification of DAI in vehicle, control, G3, G7, R3 and R8 groups. (C) Body weights. (D) Colon Images. (E) Quantification of colon length. *p<0.05; **p<0.01, compared with the vehicle group; #p<0.05; ##p<0.01, compared with the control group.

A meta-analysis suggested that tea consumption was inversely associated with UC risk (29). In mice, however, an overdose of green tea polyphenols not only failed to suppress colonic colitis but instead enhanced its formation (30). To determine the effective dosage of polyphenols in tea that confers protective effect against DSS-induced colitis, mice were pretreated with G3, G7, R3, or R8 for four days and then challenged with DSS for seven days (Figure 3A).

In all the DSS-treated groups, G7 resulted in a slight linear decrease in body weight, but all groups experienced negligible changes. (Fig. 3B). The DAI score for the control group was significantly higher at the end of the experiment than that in the tea-treated groups (Fig. 3C). The mice in the tea-treated groups exhibited regular stool consistency compared with mice in the DSS only (control) group. The teas also partially prevented rectal bleeding. Colon shortening is a visual index that reflects the severity of colorectal inflammation 31. The colorectal length of the control groups was shorter than that of the vehicle group (Fig. 3D and 3E). Administration of teas showed a preventive effect against colon shortening, although shortening remained significant. The weight of the other organs exhibited no changes.

**Figure 4:**
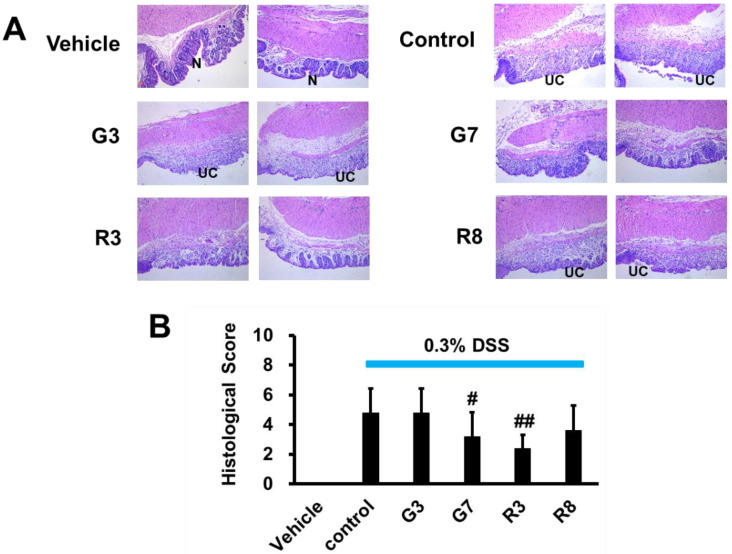
Effects of tea on colitis production in DSS-induced mice (magnification 100x). (A) Histological images of normal mucosa (N) and ulcerative colitis (UC) in vehicle, control, G3, G7, R3 and R8 with 2 mice in each group. (B) The statistical analysis of histological score. #p<0.05; ##p<0.01, compared with the control group.

We next analyzed whether teas could block the histopathological changes of the colon tissue. The H&E staining of colon tissues from the vehicle group demonstrated intact mucosal epithelia and a normal number of goblet cells (Figure 4A). In contrast, DSS-treated mice displayed significant epithelial damage, crypt distortion, and inflammatory cell infiltration in both the mucosa and submucosa. The DSS control group exhibited considerable mucosal structure damage, as demonstrated by the histologic score (Figure 4B). Among the four tea samples tested, only G7 and R3, which contained 0.021% polyphenol content, were effective in preserving the colorectal crypt structure and reducing the severity of inflammation associated with ulcerative colitis (UC) when compared to the control group and the other tea samples G3 (0.039%) and R8 (0.005%). The histological scores for G7 and R3 were also significantly lower (Figure 4B), indicating a strong inhibitory effect on DSS-induced inflammation in the distal colonic crypts and prevention of ulcerative progression. These findings suggest that G7 and R3 tea samples, with their optimal polyphenol concentrations, may have potential therapeutic benefits for those suffering from UC.

## Discussion

In this study, the toxicity and protective effects of 16 black and green teas from Taiwan were assessed using Schwann cells and H9c2 cells as *in vitro* models for preclinical evaluation of neurological and cardiovascular toxicity. The findings of the study indicated that the degree of toxicity exhibited by the teas relied on their polyphenol content, with certain concentrations of these compounds demonstrating protective effects in the cell culture models. These concentrations were observed to range between 0.0004% and 0.0011% of the total polyphenol content. Polyphenols, when present in low concentrations in the bloodstream, have been observed to generate H_2_O_2_ 9. This interaction with H_2_O_2_ can have implications for various cellular processes, including cell signaling, adaptation, and overall cell survival. It is important to note that the consumption of polyphenols in appropriate quantities and at the right timing, particularly during meals, can lead to a synergistic effect in maintaining a balanced redox state within the body.

However, it is worth noting that when the concentration of polyphenols in the blood system reaches high levels, they generate a relatively high concentration of H_2_O_2_ and potentially other derivatives. Previous research has shown that beverages rich in polyphenols, including green tea, black tea, and coffee, generate H_2_O_2_ under most physiological conditions, which may induce mutagenicity *in vitro* 18. Additionally, studies involving healthy volunteers have shown that coffee consumption leads to elevated H_2_O_2_ levels in urine 32, 33. However, the total excreted H_2_O_2_ equivalent levels in urine are estimated to account for only 0.5-10% of the consumed coffee, suggesting that the remaining H_2_O_2_ may be retained or consumed within the body 34, 35, and given that H_2_O_2_ in the body is associated with carcinogenicity and genotoxicity 36, reducing H_2_O_2_ formation in beverages is desirable.

In H9C2 cardiomyocytes, it was observed EGCG displayed a time- and dose-dependent inhibition of proliferation and induction of apoptosis 37, in which the involvement of SIRT1, a protein associated with cellular processes, was identified. It was suggested that high doses of tea polyphenols, such as EGCG, may be toxic for cardiomyocytes and nerve cells, and related to the inhibition of the SIRT protein. The results from this study demonstrates the effects of oxidative stress from polyphenols which contain H_2_O_2_-generating properties.

Colorectal cancer (CRC) is highly associated with environmental factors and geographical cultural conditions (e.g., gender, race, and lifestyle) and is currently the fifth leading cause of cancer-related deaths worldwide 38, 39. Epidemiological research has identified diet as a major factor influencing CRC risk 40. Diets high in protein, fat, and refined carbohydrates may disrupt the balance of microbes in the colon, promoting the progression of inflammatory bowel disease (IBD) and increasing CRC risk 41-43. Therefore, suppressing inflammation is an important strategy in cancer prevention. Dextran sulfate sodium (DSS) is known to induce ulcerative colitis (UC) by damaging cell membranes, increasing colonic epithelial permeability, and altering tight junction protein expression 44, 45. Murakami reported that diets containing low doses (0.01-0.1%) of green tea polyphenols reduced DSS-induced colitis in experimental animal studies, however, diets containing high doses (0.5-1%) of green tea polyphenols aggravated the colitis formation 46. Therefore, this study utilized DSS-induced UC as an *in vivo* model. The results showed that although tea extracts did not restore colon length, G7 and R3 alleviated inflammation, while G3 and R8 did not, being at an ineffective dosage. This observation, combined with the known antioxidant and anti-inflammatory properties of polyphenols, led researchers to conclude that tea extracts may not inhibit DSS-induced damage to the colonic mucosa but can prevent DSS-induced colitis inflammation in the moderate dosage.

To determine the optimal tea consumption, *in vivo* and *in vitro* studies were conducted, emphasizing the importance of polyphenol content in black and green teas. For example, if the polyphenol content in G7 or R3 (10g/100ml) is 0.14% or 0.07% respectively, the ideal consumption for a 50 kg person would be 500 ml of hot water (containing 0.7 g G7 or 1.6 g R3) to achieve a protective prophylactic concentration of polyphenols (0.2 mg/kg) 47. These emphasize the importance of determining the polyphenol content in black and green teas to establish the most beneficial consumption amounts 48.

## Conclusion

**Figure 5:**
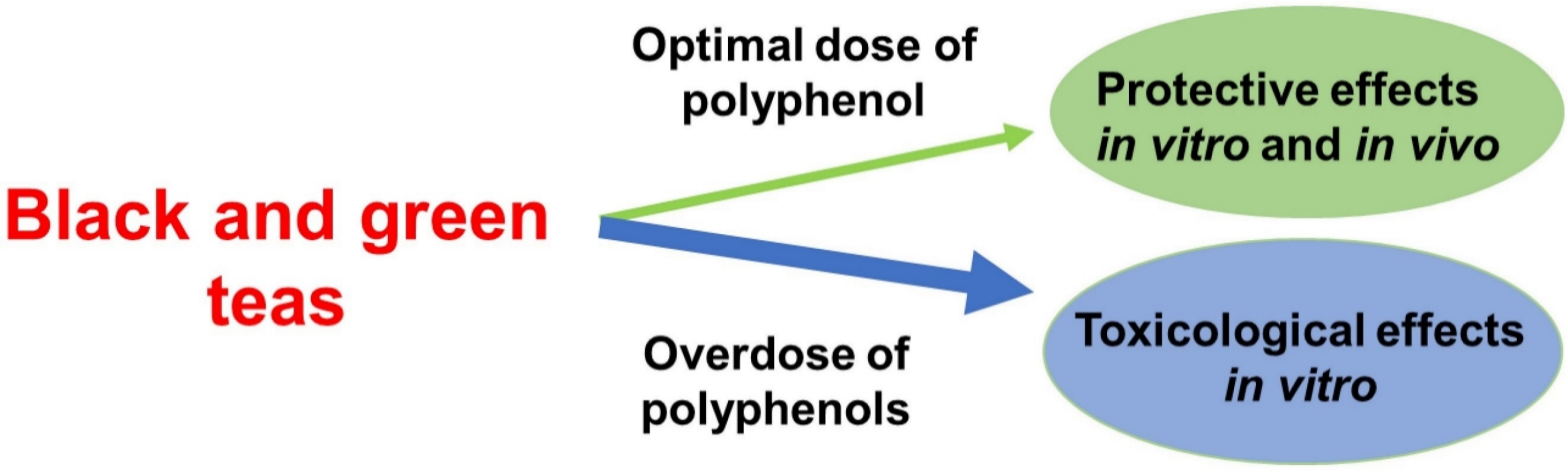
The protective and toxicity effects of black and green teas. Consuming an optimal polyphenol dose of teas was found to be more protective effects *in vivo* and *in vitro,* as well as consuming an overdose of polyphenols to be toxicological effects. This suggests that the efficacy of teas in treating inflammation-induced diseaases is influenced by the amount of polyphenols consumed.

This research highlights the potential oxidative effects of polyphenols both *in vitro* and *in vivo*, as well as their role in mitigating inflammation in DSS-induced colitis, and shows that, to account for the toxicity as well as protective effect of black and green tea, the optimal concentration in cell culture models range between 0.0004% and 0.0011% of polyphenol content (Figure 5). These research findings can serve as guidelines for determining the optimal tea consumption based on polyphenol content and individual body weight, ultimately promoting health benefits and reducing potential risks associated with tea consumption.

## Figures and Tables

**Table 1:** The IC50 (mg/ml) toxicity of green and black teas *in vitro*. The unit of IC50 were adjusted to polyphenolic concentration (pIC50) (% of polyphenol,). Sixteen commercial tea samples belonging to the species of *Camellia sinensis* including green teas (G1-G8, eight samples) and black teas (R1-R8, eight samples) from central Taiwan were collected. Each tea sample (10 g) was soaked in 100 ml boiled distilled water for 10 min. The polyphenols were detected by Folin-Ciocalteu method.

Tea	Crude leave extracts (% of polyphenols)	RSC96 (IC_50_) (mg/ml)	RSC96 (pIC_50_) (% of polyphenols)	H9c2 (IC_50_) (mg/ml)	H9c2 (pIC_50_) (% of polyphenols)
G1	0.16	2.73	0.0085	1.11	0.0035
G2	0.15	3.24	0.0098	1.34	0.0040
G3	0.26	2.03	0.0104	0.90	0.0046
G4	0.18	2.67	0.0094	1.05	0.0037
G5	0.25	1.87	0.0095	0.67	0.0034
G6	0.21	2.07	0.0088	0.99	0.0042
G7	0.14	3.10	0.0087	1.20	0.0034
G8	0.26	2.10	0.0110	1.05	0.0055
R1	0.06	5.82	0.0075	3.34	0.0043
R2	0.10	5.79	0.0118	1.78	0.0036
R3	0.07	9.49	0.0142	3.00	0.0045
R4	0.13	3.08	0.0083	1.13	0.0030
R5	0.16	2.47	0.0080	1.07	0.0035
R6	0.17	2.43	0.0085	0.91	0.0032
R7	0.04	10.38	0.0091	2.37	0.0021
R8	0.03	15.26	0.0097	3.66	0.0023
